# Recalibration of Framingham risk for a local population of Sri Lanka

**DOI:** 10.1186/s12889-023-17601-8

**Published:** 2024-01-12

**Authors:** Sameera Upashantha Ranasinghe, E. M. D. S. Ekanayake, Laksitha Iroshan Ranasinghe, Sampath U. B. Tennakoon

**Affiliations:** 1 Ministry of Health, Nutrition and Indigenous Medicine, Colombo, Sri Lanka; 2 Faculty of Medicine, University of Peradeniya, Peradeniya, Sri Lanka; 3 Family Health Bureau, Colombo, Sri Lanka

**Keywords:** Cardiovascular diseases, Risk prediction, Framingham equation, Sri Lanka, Recalibration

## Abstract

**Background:**

Cardiovascular Diseases (CVD) account for the highest number of deaths and disability globally and within Sri Lanka. A CVD risk prediction tool is a simple means of early identification of high-risk groups which is a cost-effective preventive strategy, especially for resource-poor countries. Distribution of risk factor levels varies in different regions even within the same country, thus a common risk estimation tool for the country may give false local predictions. Since there are few published data related to Sri Lanka the aim of this study was to recalibrate the Framingham equation according to the local risk factor profile of a population in the Kurunegala region in Sri Lanka.

**Method:**

A cross-sectional study was conducted with the participation of 1 102 persons from the Kurunegala Regional Director of Health Services area and the data was collected using an interviewer-administered questionnaire, anthropometric, blood pressure, and biochemical measurements. CVD risk was estimated using Framingham original and recalibrated CVD risk assessment methods. Current CVD mortality and morbidity data and the recalibration method conducted by the method described by Wilson and colleagues were used for calculations.

**Results:**

Original and recalibrated Framingham CVD risk scores predicted 55.5% (*N* = 612) and 62.3% (*N* = 687) to be having less than 10% CVD risk respectively. Further, the original and recalibrated CVD Risk Scores predicted 2.2% (*N* = 24) and 1.8% (*N* = 20) to be having CVD risk more than 40% respectively.

**Conclusion:**

These findings show an over prediction of the CVD risk with the original Framingham risk calculations which signifies the importance of development of a region-specific risk prediction tool using local risk factor data in Sri Lanka which will prevent unnecessary expenditure to manage people without risk of CVD.

**Supplementary Information:**

The online version contains supplementary material available at 10.1186/s12889-023-17601-8.

## Introduction

Out of the non-communicable Diseases (NCD), cardiovascular diseases (CVD), mainly; ischemic heart disease (IHD) and Cerebrovascular Diseases (CVD were the leading causes of death and a top cause for Disability Adjusted Life Years (DALYs) for both sexes in 2019 globally [1]. In Sri Lanka, case fatality rates of IHD and CVD were as high as 5.58 and 6.78 per 100 cases in 2019 [2] while consuming one third of the health expenditure in 2017 and 2018 [3]. Being the fastest ageing country in south Asia, with a projected 22.4% elderly by 2030, will most definitely contribute to worsening the situation [4]. Although globally applicable multiple risk factors have been identified for CVD, the risk factor profile may differ by the region of residence/ethnicity [5, 6]. South Asians are known to have higher levels of risk factors such as lack of fruit and vegetable consumption and lack of engagement in physical activity compared to individuals from other regions [7]. South Asians had high levels of total and Low-Density Lipoprotein (LDL) Cholesterol, triglycerides, glucose intolerance and low High-Density Lipoprotein (HDL) Cholesterol levels than other ethnic groups [8–10]. This was an observation among members of the same family residing in different regions of the world [11]. Sri Lanka being a south Asian country a similar risk profile to many other countries of the region has been identified. 72.5% of the population was not consuming five servings of fruits and vegetables daily, 30.1% was not having sufficient physical activities, 15.0% was smoking and 17.9% was taking alcohol in addition to the similarities with countries of the region [12].

When it comes to prevention of CVD, the total risk approach based on multiple risk factors is proven to be effective and cost effective, especially for low-income countries The Framingham Risk Equation is one such total risk assessment tool developed to calculate CVD risk through the Framingham cohort study. The calculation takes in to account age, sex, smoking status, total Cholesterol level, HDL Cholesterol level, Systolic Blood Pressure (SBP), presence of Diabetes Mellitus and stage of hypertension [13]. Although the Framingham risk assessment is accurate for the population for which it was developed it has been found to overestimate the risk among people of non-European origin [14–16].

Hence, the main objective of this study was to recalibrate the Framingham risk equation with local risk factor levels of residents of Kurunegala Regional Director of Health Services (RDHS) area in Sri Lanka.

## Methods

This was a cross sectional analytical study carried out between 31^st^ of August 2019 to 31^st^ of April 2020 in the Kurunegala RDHS (the country is divided in to 25 regional director of health services areas for administrative functions of health care provision within a district) of Sri Lanka, which is the third most populated RDHS area in Sri Lanka. Thirty- to 59-year-old registered residents who had lived a minimum of one year, within the area selected was the study population. The 1-year minimum period was to reduce the bias that can occur from internal migration of population. Further, the age selection was aimed at determining any CVD risk trends in the population less than 35 years of age which, is the lower limit of the national NCD screening program of Sri Lanka. Upper age limit was selected as less than 60 years to predict all the premature mortality that can occur due to CVD. Those who were suffering from CVD endpoints (Myocardial Infarction, Stroke, Congestive Heart Failure, Coronary Artery Bypass Surgery, Coronary Angioplasty and on treatment for Angina) were excluded. Those who have been already followed up by Healthy Life-style Centres were excluded as well, as their risk factor levels might have been changed due to behavioural modifications.

The sample size was calculated using the following formula [17].

$$N=\frac{{\left(Z\alpha \right)}^{2}*P\left(1-P\right)*DE}{{d}^{2}}$$

where Z_α_ was the level of statistical significance (3.92), P was the expected proportion of high CVD risk (7%), precision at 0.025 and the design effect was considered as 2. Minimum sample required was 960 with an inflation by 20% for contingencies. Stratified 3 stage Random Sampling was employed to select the study participants. In the first stage, 10 out of the total 29 Medical Officer of Health areas which, are the grass root level health units providing preventive care in Sri Lanka, of the Kurunegala RDHS area were chosen.

In stage 2, out of the 10 MOH areas, three Public Health Midwife (PHM) areas were randomly selected. The average population of a PHM area is 3000. In stage 3, the required number of participants were recruited proportionate to the size of the population of respective PHM area through interval sampling. From each PHM area, approximately similar number of participants (32) were selected from each age stratum of 30–39, 40–49 and 50–59 years as per the distribution of Sri Lankan general population [18]. Within the selected household, if more than one person was living within 30–60-year age limit, the recruitment was done using Simple Random selectionn. However, due to the requirements of the practical settings and the sustainability of the project 1102 participants (632 females and 470 males) were investigated. Data collection was carried out with the informed written consent of the participants by principal investigator and two trained and qualified assistants. Data was collected through an interviewer administered questionnaire, anthropometric measurements, blood pressure measurements and biochemical analysis of a blood sample of the participants. To capture the working population the data collection was carried out on public holidays and weekends with prior notification. The data collection during the times of cultural celebrations (New Year, Christmas) was withheld to reduce the bias imposed by changes in the lifestyle. The blood collection was done at a common venue in the selected Public Health Midwife area by two trained phlebotomists under the supervision of the principal investigator.

When considering socio demographic and behavioral risk data; age, sex, smoking status, alcohol consumption, fruit and vegetable consumption and physical activity level were recorded. Smoker was defined as a person who has smoked at least a single cigarette in the past six months [19]. Alcohol consumption was defined as consuming at least one drink of alcohol in the past 30 days [20]. Additionally, family history of Cardiovascular Endpoints (Myocardial Infarction, Stroke, Congestive Heart Failure, Coronary Artery Bypass Surgery, Coronary Angioplasty, on treatment for Angina) in first degree relatives was recorded. Data related to fruit and vegetable consumption was recorded as a 24 h recalll and the cut off values were assigned according the guidelines [21]. Physical activity level of all the participants were assessed using interviewer administered physical activity questionnaire (short International Physical Activity Questionnaire). The cut off values were assigned according to standards [22]. The predicted CVD risk of each of the participants were calculated using Framingham original and recalibrated methods One of the limitation of the study was not using participants above 70 years for the screening purposes.

### Data entry and analysis

Data was entered into SPSS 25 program for analysis. Five percent of the data was double entered to look for errors in data entry and there were no errors detected.

### Recalibration of Framingham equation

The CVD risk was calculated using the method described by Wilson and colleagues [23]. Beta coefficients of the Cox Regression, local 10 year CVD Event Rate and Local Mean Risk [24].

#### Step 1

Original Cox Regression Coefficients from the Framingham Study (Supplementary material Table 1) and the risk factor levels from the current study (Supplementary material Table 2) were used to form the recalibrated equation. The risk factor levels of the original Framingham study were substituted with local risk factor data to form the new recalibrated equation. The result from the equation was stored in a function called B_i M_i. This function was calculated for both males and females [25].

**Table 1:** Distribution of study participants by socio demographic and cardiovascular risk factors

**Variable**		**N (%)**	**Age (mean, SD)**
**Sex**	Female	632 (57.4)	47 years (9.1)
	Male	470 (42.6)	45.8 years (8.8)
**Age Category(years)**	30—39	363 (32.9)	
	40—49	369 (33.5)	
	50—59	370 (33.6)	
**Family history of Cardiovascular Diseases**	No	717(65.1)	
	Yes	385 (34.9)	
**On Antihypertensive medications**	No	796 (72.2)	
	Yes	306 (27.8)	
**Physical activity level**	High	446 (40.5)	
	Moderate	452 (41.0)	
	Low	204 (18.5)	
**Sedentary time per week**	High (> = 14 h per week)	474 (43.0)	
	Low (< 14 h per week)	628 (57.0)	
**Daily adequate fruits and vegetable consumption**	Yes	358 (32.5)	
	No	744 (67.5)	
**Alcohol consumption within past 30 days**	Yes	325 (29.5)	
	No	777 (70.5)	
**Smoking within the past six months**	Yes	141 (12.8)	
	No	961 (87.2)	
**Blood Pressure stage**	Normal (< 120/80 mmHg)	335 (30.4)	SBP-124.9 mmHg (19.9) DBP-80.3 mmHg (19.9)
	Elevated (120–129/ < 80 mmHg)	257 (23.3)	
	Stage 1 Hypertension (130–139/80-89 mmHg)	199 (18.1)	
	Stage 2 Hypertension (> = 140/ > = 90 mmHg	311 (28.2)	
**Total Cholesterol levels (mg/dl)**	< 160	149 (13.5)	209.9 mg/dl (46.5)
	160–199	303 (27.5)	
	200–239	392 (35.6)	
	240–279	188 (17.1)	
	> 280	70 (6.4)	
**HDL Cholesterol levels (mg/dl)**	< 35	33 (3.0)	51.7 mg/dl (8.7)
	35–44	158 (14.3)	
	45–49	163 (14.8)	
	50–59	608 (55.2)	
	> 60	140 (12.7)	
**LDL Cholesterol levels (mg/dl)**	< 100	280 (25.4)	128 mg/dl (41.8)
	100–129	291 (26.4)	
	130–159	308 (27.9)	
	160–189	149 (13.5)	
	= > 190	74 (6.7)	
**Triglyceride levels (mg/dl)**	< 150	794 (72.1)	134.5 mg/dl (57.1)
	150–199	184 (16.7)	
	200–499	123 (11.2)	
	= > 500	1 (0.1)	
**Fasting blood sugar**	High^a^	161 (14.6)	92.8 mg/dl (37)
	Normal	941 (85.4)	

^a^>  = 126 mg/dl

**Table 2:** Cardiovascular Diseases Risk Predicted by Framingham Cardiovascular Diseases Scores

		Original Framingham CVD risk scores	Recalibrated Framingham CVD risk scores	Mean differences between Framingham Original Cardiovascular Diseases Score and Recalibrated Framingham Cardiovascular Diseases Score
N	Valid	1102	1102	Mean difference = 3.64 ( T value = 40.9,df = 1101, *p* < 0.01)
	Missing	0	0	
Mean		11.77%	8.13%	
Standard Deviation		10.18	8.13	
Minimum		0.16%	0.13%	
Maximum		78.52%	70.50%	

B_i M_i Function for females;




B_i M_i Function for males.




#### Step 2

A function named B_i X_i was calculated for each individual participant using each individual risk factor level.

B_i X_i Function for female;




B_i X_i Function for male;




#### Step 3

For each individual, the function B_i M_i calculated at step one was deducted from function B_i X_i to produce a function called A.

$$\mathrm A\;=\;\mathrm{B_i}\;\mathrm X\;\mathrm{_i}\;-\;\mathrm{B_i}\;\mathrm{M_i}$$

Then exponential of A was taken as B

$$\mathrm B=\;\mathrm e^\wedge\mathrm A$$

#### Step 4

Then the 10-year probability of CVD Event Rate (P) was calculated using CVD Free Survival Rate (S(t)) for Kurunegala Regional Director of Health Services area, calculated using the area specific CVD Mortality and Morbidity Data in a similar crude method conducted at Indian recalibration study [23, 26].

$$\begin{array}{ll}\mathrm{CVD}\;\mathrm{events}\;(\mathrm{fatal}\;\mathrm{and}\;\mathrm{non}-\mathrm{fatal})\;\mathrm{number}\;\mathrm{in}\;\mathrm{males}\;\mathrm{in}\;2019\;\mathrm{in}\;\mathrm{Kurunegala}&=\;9835\\\mathrm{CVD}\;\mathrm{events}\;(\mathrm{fatal}\;\mathrm{and}\;\mathrm{non}-\mathrm{fatal})\;\mathrm{number}\;\mathrm{in}\;\mathrm{females}\;\mathrm{in}\;2019\;\mathrm{in}\;\mathrm{Kurunegala}&=7465\\(\mathrm{Statistics}\;\mathrm{Officer},\;2020)&\\\mathrm{Male}\;\mathrm{population}\;\mathrm{in}\;\mathrm{Kurunegala}\;2019&=\;838351\\\mathrm{Female}\;\mathrm{population}\;\mathrm{in}\;\mathrm{Kurunegala}\;2019&=\;855460\end{array}$$

$$\begin{array}{ll}\mathrm{CVD}\;\mathrm{event}\;\mathrm{rates}\;\mathrm{in}\;\mathrm{males}&=\;(\mathrm{number}\;\mathrm{of}\;\mathrm{events}\;\mathrm{in}\;\mathrm{males})/(\mathrm{population})\\&=\;9835\;/\;838351\\&=\;0.011802\end{array}$$

$$\begin{array}{ll}\mathrm{CVD}\;\mathrm{free}\;\mathrm{survival}\;\mathrm{rate}\;\mathrm{of}\;\mathrm{males}(\mathrm S(\mathrm t))&=1-0.011802\\&\;=\;{(0.988198)}^{10}\;=\;0.8881\end{array}$$

$$\begin{array}{lc}\mathrm{CVD}\;\mathrm{event}\;\mathrm{rates}\;\mathrm{in}\;\mathrm{females}&=\;(\mathrm{number}\;\mathrm{of}\;\mathrm{events}\;\mathrm{in}\;\mathrm{females})\;/\;\mathrm{population}\\&=\;7465\;/\;855460\\&=\;0.008726\end{array}$$

$$\begin{array}{ll}\mathrm{CVD}\;\mathrm{free}\;\mathrm{survival}\;\mathrm{rate}\;\mathrm{of}\;\mathrm{females}\;(\mathrm S(\mathrm t))&=1-0.008726\\&={(0.991274)}^{10}\;=\;0.9161\end{array}$$

$$P=\;1\;-\;\left[S\;\left(t\right)\right]^B$$

## Results

Daily adequate consumption of fruits and vegetables was low among most of the participants (*N* = 328, 32.5%). Majority of the participants (*N* = 392,35.6%) has had total cholesterol levels between 200–239 mg/dl (Table 1).

**Fig. 1:**
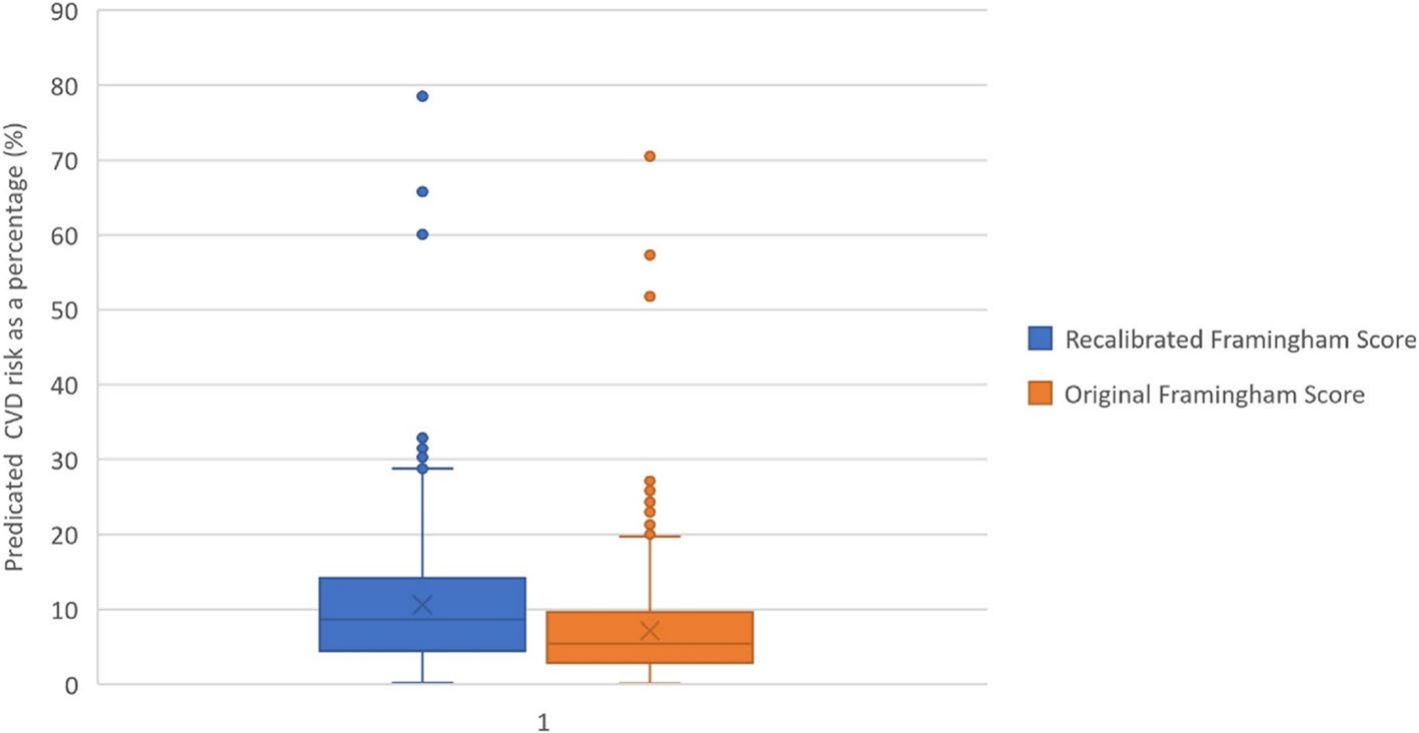
Distribution of CVD risks predicted by recalibrated and Original Framingham Scores

Original Framingham CVD Risk Scores produced a mean ten-year risk of 11.7% while Recalibrated Framingham CVD Risk Scores produced a mean of 8.13% (Table 2). The CVD risks calculated by Original and Framingham CVD risk scores are distributed with medians of 8.6% and 5.4% (Fig. 1). Maximum CVD risk predicted by both scores were approximately 70%. A statistically significant difference was observed between CVD risk by Framingham Original CVD Risk Score and Recalibrated Framingham CVD Risk Score (t = 40.9, df = 1 101, *p* < 0.001) (Table 2).

Highest percentage of females were marked as having > 20% CVD risk by the Original Framingham Score (*N* = 87,13.8%) followed by recalibrated Framingham Score (*N* = 57,9%). A similar pattern was observed among males, where 21.3% % (*N* = 100) was categorized into high CVD risk by Original Framingham Scores followed by recalibrated Framingham Score (*N* = 63, 13.4%). There were significant differences by sex in categorization in to > 20% CVD risk in recalibrated Framingham Method (9% females vs. 13.4% males, *p* = 0.02) and original Framingham Method (13.8% females vs. 21.3%% males, *p* = 0.01) (Table 3).

**Table 3:** Distribution of CVD risk according to sex

		**Sex**				***P*** ** Value**
		**Female**		**Male**		
		**N**	**%**	**N**	**%**	
**Original Framigham score**	> 20%risk	87	13.8%	100	21.3%	0.01
	< 20risk	545	86.2%	370	78.7%	
	Total	632	100.0%	470	100.0%	
**Recalibrated Framigham score**	> 20%risk	57	9.0%	63	13.4%	0.021
	< 20%risk	575	91.0%	407	86.6%	
	Total	632	100.0%	470	100.0%	

Framingham Original CVD Risk Score categorized the highest percentages of individuals (15.2%, *N* = 56,34.9%, *N* = 129) into high CVD risk in the 40–49- and 50–59-years age groups compared to other methods. Recalibrated Framingham CVD Risk Score categorized the highest percentage of individuals (1.9%, *N* = 7) into high CVD risk in the 30–39 years age group. in 30–39, 40–49- and 50–59-years age groups respectively (Table 4).

**Table 4:** Distribution of cardiovascular diseases risk according to age groups

		**Age Category**						
		**30–39**		**40–49**		**50–59**		
		**Count**	**%**	**Count**	**%**	**Count**	**%**	***P*** ** value**
**Original Framigham score**	> 20%risk	2	0.6%	56	15.2%	129	34.9%	< 0.01
	< 20risk	361	99.4%	313	84.8%	241	65.1%	
	Total	363	100.0%	369	100.0%	370	100.0%	
**Recalibrated Framigham score**	> 20%risk	7	1.9%	18	4.9%	95	25.7%	< 0.01
	< 20%risk	356	98.1%	351	95.1%	275	74.3%	
	Total	363	100.0%	369	100.0%	370	100.0%	

Original and recalibrated Framingham CVD risk scores predicted 55.5% (*N* = 612) and 62.3% (*N* = 687) to be having less than 10% CVD risk respectively. Further, the original and recalibrated CVD Risk Scores predicted 2.2% (*N* = 24) and 1.8% (*N* = 20) to be having CVD risk more than 40% respectively (Table 5).

**Table 5:** Percentage distribution of predicted cardiovascular diseases risk categories by selected risk estimate methods

Risk category	Original Framingham CVD score		Recalibrated Framingham CVD score	
	N	%	N	%
< 10%	612	55.5%	687	62.3%
> = 10%- < 20%	303	27.5%	295	26.8%
> = 20%- < 30%	124	11.3%	76	6.9%
> = 30%- < 40%	39	3.5%	24	2.2%
> = 40%	24	2.2%	20	1.8%

## Discussion

Original Framingham Risk Scores identified 17% as having high CVD risk >  = 20% (Table 5). This is extremely low when compared to Mettananda et al. (mean HDL 48.58mg/dl, CVD risk > 20% = 36.7%, age of the sample 48–78 years) [27] and Ranawaka et al. (mean HDL- 23.4mg/dl, CVD risk > 20% = 37.2%, age of the sample-35–65 years) [28]. Studies from Sri Lanka. The reason can be attributed to the high HDL Cholesterol level found in this study compared to other two studies. HDL Cholesterol level directly affects the measurement of Framingham Original CVD Risk score. High HDL Cholesterol levels lower the CVD Risk calculated with the Framingham Risk Scores CVD Equation. Further, the age groups used in those studies were older compared to the present study. Approximately one third of the participants in this study were in the 30–39 years age group and none were above or equal to 60 years. This might have resulted in these low percentages of high CVD Risk by original version of the Framingham Risk Scores in the present study. Further, the percentage on antihypertensive in Mettananda et al. study was higher than 27.8% that was found in the present study. The Ranawaka et al. study reported 2.7% with previous CVD events who were included in the risk calculation. When comparing with studies from other parts of the world, the proportions with high CVD risk categories are low in the present study [29–32]. High hypercholesterolemia percentages and low mean HDL Cholesterol in these studies might have resulted in the said differences. High mean HDL Cholesterol level (Table 5) in the present study provides a large negative value on BiXi function. Accordingly, it results in a large reduction in CVD risk.

In the present study, Framingham recalibrated version predicted a total of 10.9% (9.0% females and 13.4% males) to be in high CVD Risk categories which was lower than the risk calculated by the original version (13.8% in females and 21.8% males). In an Australian Indigenous study, the recalibrated Framingham Risk Scores returned a higher risk of 19.6% and 22.9% compared to 8.9% and 15.4% with the original version for males and females respectively [15]. In a Hong Kong study, recalibrated Framingham Risk Scores had predicted 36.1% males and 22.2% females to be in the high CVD Risk category which was more or less similar to the risk scores by the original version [33]. In the Hong Kong study, the population was older (mean 65 years) which may have given rise to the higher high CVD risk proportions. Although the Australian Indigenous study had a similar lower age cut-off the upper end was 74 as against the 60 years of the current study which may be the reason for the higher risk category being larger. In interpreting these results we have to take into consideration that the recalibration was conducted using beta coefficients of the Framingham Original Study replacing only the local risk factor mean values and CVD survival rate data. Comparatively different mean values and survival data might be producing different findings. Especially, when compared to these studies the proportion smoking among females is zero in the present study. The smoking has a higher impact on the equation itself. Therefore, the different results might have been obtained. When calculating the BiMi function the high mean of HDL Cholesterol level in the present study might have also led to low CVD Risk levels in the final calculation in the present study (refer-methodology section).

There was a significant difference between the mean CVD risk values of both methods. When considering low CVD risk strata, percentage agreements between the scores were higher compared to high CVD risk categories. The reason might be the different mean values of risk factors and survival rates used in the recalibration than Framingham Original population. In the present study Original Framingham CVD Risk Equations predicted significantly higher proportion of males than females to have high CVD risk (21.3% vs 13.8%). Similarly in Mettananda et al. study and in an Iranian study Framingham Original CVD Risk Score predicted significantly high proportion of males in to high CVD risk category [27, 34]. High dyslipidaemia levels among males in these studies might have resulted in these high CVD risk calculations [35].

The follow-up study has been planned to assess the predictive capacity of the Framingham function and other CVD risk assessment methods. It has been planned to contact participants with regard to the development of CVDs at the 10 years of time. For healthy proportion of the participants, another screening similar to the present study, to assess the CVD risks in similar manner.

## Conclusions

These findings show that the original Framingham score overestimates the 10-year CVD risk compared to the recalibrated Framingham risk prediction equation in Sri Lanka, which reinforces the importance of development of region-specific risk prediction tools or re-calibrating existing tools using local risk factor data from Sri Lanka which will prevent unnecessary expenditure to manage people without risk and neglecting people with risk.

### Supplementary Information

**Additional file 1. **

## Data Availability

All the raw data and material are available with the corresponding author to be produced at request.
