# Supplementary material for: Recalibration of Framingham risk for a local population of Sri Lanka
Source: BMC Public Health. 2024 Jan 12;24:165. doi: 10.1186/s12889-023-17601-8 (PMC10785388; doi:10.1186/s12889-023-17601-8)
Supplement: Supplementary file 1 — Additional file 1. [file 12889_2023_17601_MOESM1_ESM.docx]

Figures and Tables

Table 1

Cox regression coefficients and 95% confidence intervals (95% CI) for the risk factors included in the Framingham functions for 10 year incidence of coronary deaths and recognized non-fatal myocardial infarction (hard end points) by sex (D'Agostino Sr et al., 2001)

| **Risk factors(units)** | **Risk factor categories** | **Men Cox regression coefficient estimates (95% CI)** | **Women Cox regression coefficient estimates (95% CI)** |
| --- | --- | --- | --- |
| Age (y) | Mean | 0.05 (0.04 to 0.07) | 0.17 (−0.03 to 0.37) |
| Age (y) squared | Mean squared |  | −0.001 (0.003 to 0.001) |
| Total cholesterol (mg/dl) | <160 | −0.38 (−1.18 to 0.42) | −0.21 (−1.73 to 1.32) |
|  | 160–199 | 0 | 0 |
|  | 200–239 | 0.57 (0.22 to 0.92) | 0.44 (−0.21 to 1.09) |
|  | 240–279 | 0.74 (0.36 to 1.13) | 0.56(-0.11 to 1.22) |
|  | >=280 | 0.83 (0.33 to 1.32) | 0.89 (0.19 to 1.80) |
| HDL Cholesterol (mg/dl) | <35 | 0.61 (0.16 to 1.06) | 0.73 (0.00 to 1.46) |
|  | 35–44 | 0.37 (−0.06 to 0.79) | 0.60 (0.05 to 1.15) |
|  | 45–49 | 0 | 0.60 (0.05 to 1.14) |
|  | 50–59 | 0.00 (−0.47 to 0.47) | 0 |
|  | >59 | −0.46 (−1.09 to 0.17) | −0.54 (−1.10 to 0.02) |
| Blood pressure  (mm Hg) | Optimal (S<80) | 0.09 (−0.41 to 0.60) | −0.74 (−1.53 to 0.05) |
|  | Normal (S 120–129)/(D 80– 84) | 0 | 0 |
|  | High normal (S 130–139)/(D  85–89) | 0.42 (−0.02 to 0.86) | –0.37 (–1.09 to 0.35) |
|  | Stage I (S 140–159)/(D90–99) | 0.66 (0.25 to 1.07) | 0.22 (−0.38 to 0.81) |
|  | Stage II–IV (S>160)/(D>100) | 0.90 (0.46 to 1.33) | 0.61 (0.00 to 1.21) |
| Diabetes |  | 0.53 (0.11 to 0.94) | 0.87 (0.33 to 1.40) |
| Smoking | Current 1 cigarette/day  consumption | 0.73 (0.47 to 0.98) | 0.98 (0.57 to 1.38) |

S, systolic blood pressure; D, diastolic blood pressure; HDL, high density lipoprotein. *Risk factor means refers to the mean age and to the proportion of population in each level of the risk factors.

Table 2 Local risk factor levels of the sample applied for the equation

|  | **Male** | **Female** |
| --- | --- | --- |
| **Age(Years)** | 45.8 | 47.09 |
| **Total Cholesterol (mg/dl)** | 207.3 | 211.85 |
| **HDL Cholesterol (mg/dl)** | 50 | 52.91 |
| **Systolic blood pressure mmHg** | 126.34 | 123.8 |
| **Percentage Treated for high blood pressure (%)** | 22.8 | 31.5 |
| **Percentage Smoking (%)** | 30 | 0 |
| **Percentage of Diabetes (%)** | 15.1 | 14.2 |

These data were applied in the recalibration.
